# Supplementation of serum albumin is associated with improved pulmonary function: NHANES 2013–2014

**DOI:** 10.3389/fphys.2022.948370

**Published:** 2022-10-03

**Authors:** Sheng Hu, Qiang Guo, Silin Wang, Wenxiong Zhang, Jiayue Ye, Lang Su, Sheng Zou, Deyuan Zhang, Yang Zhang, Dongliang Yu, Jianjun Xu, Yiping Wei

**Affiliations:** Department of Thoracic Surgery, The Second Affiliated Hospital of Nanchang University, Nanchang, China

**Keywords:** serum albumin, pulmonary function, FVC, FEV 1, NHANES

## Abstract

**Background:** The serum albumin level is reflective of the function of multiple organs, such as the liver and kidneys. However, the association between serum albumin and pulmonary function is unclear; therefore, this study aimed to determine the relationship between pulmonary function and serum albumin, including the threshold of serum albumin at the changes of the pulmonary function in the total population and in different strata of population.

**Methods:** In this cross-sectional study, We examined the relationship between serum albumin and two independent indicators of pulmonary function: forced vital capacity (FVC) and forced expiratory volume in one second (FEV 1), using data from National Health and Nutrition Examination Survey (NHANES 2013–2014) (n = 3286). We used univariate analysis, stratified analysis, and multiple regression equation analysis to examine the correlation between serum albumin levels and FVC and FEV 1, and performed smoothed curve fitting, threshold effect, and saturation effect analysis (for stratification) to determine the threshold serum albumin level at which FVC and FEV 1 begin to change.

**Results:** The adjusted smoothed curve fit plot showed a linear relationship between serum albu-min levels and FVC: for every 1 g/dl increase in the serum albumin level, FVC increased by 80.40 ml (11.18, 149.61). Serum albumin and FEV 1 showed a non-linear relationship. When serum al-bumin reached the inflection point (3.8 g/dl), FEV 1 increased with increasing serum albumin and the correlation coefficient β was 205.55 (140.15, 270.95).

**Conclusion:** Serum albumin is a core indicator of liver function, and abnormal liver function has a direct impact on pulmonary function. In the total population, serum albumin levels were linearly and positively correlated with FVC. Above 3.6 g/dl, serum albumin was positively correlated with FEV 1. Based on the total population and different population strata, this study revealed a positive association between the serum albumin level and pulmonary function, and identified the threshold of serum albumin when Indicators of pulmonary function tests starts to rise, providing a new early warning indicator for people at high risk of pulmonary insufficiency and has positive implications for the prevention of combined respiratory failure in patients with liver insufficiency.

## 1 Introduction

The National Health and Nutrition Examination Survey (NHANES) database is a rigorous and comprehensive database, and many influential studies have been derived from NHANES data. For example, Chen et al. (Chen et al., 2019) used the NHANES database to study the relationship between weight change and mortality in American adults. Pulmonary function tests are valuable in the treatment of patients with suspected or previously diagnosed respiratory disease. They help with diagnosis, monitoring the response to therapy, and guiding decisions regarding further treatment and intervention. Forced vital capacity (FVC), forced expiratory volume in one second (FEV 1), and other parameters have been used in the diagnosis and monitoring of respiratory disorders, such as chronic obstructive pulmonary disease (COPD), interstitial lung disease, and asthma (Kazemi, 1968; Wells, 2007; Mohamed Hoesein et al., 2011; Johnson and Theurer, 2014; Dempsey and Scanlon, 2018; Langan and Goodbred, 2020). However, pulmonary function tests might be contraindicated in some cases, such as, bullae, severe chest trauma, within 1 month of myocardial infarction, and in patients in an intensive care unit (ICU). In addition, pulmonary function tests are routinely performed before thoracic surgery; however, they are only one-time tests, and there is no ideal dynamic method to monitor pulmonary function, because 3 weeks after surgery is also a contraindication for pulmonary function tests (Cooper, 2011). Therefore, the current and commonly used pulmonary function tests are sometimes inadequate to support decisions regarding necessary respiratory support. Therefore, we wondered if there is a better way to solve these problems. We examined many data and literature and found that the serum albumin level might be a good indicator.

The serum albumin level is the main determinant of plasma colloidal osmotic pressure (Coward, 1975; Caraceni et al., 2013), and is a possible indicator of general physical health. Physiologically, serum albumin has the functions of binding and transporting endogenous and exogenous substances, scavenging free radicals, anti-oxidation, inhibiting platelets, anti-coagulation, and affecting the permeability of arteries and blood vessels (Roche et al., 2008; Fanali et al., 2012; Infusino and Panteghini, 2013; Lam et al., 2013; Wang et al., 2015; Sun et al., 2019). Serum albumin is closely related to multiple organ functions, such as the liver and kidney, which are closely related to pulmonary function.

A study involving 35 participants with COPD and cachexia showed that serum albumin levels were independently related to FVC and FEV 1 values (Görek Dilektaşli et al., 2009). However, in the general healthy population, or the larger population, this has not been studied so far. Therefore, we believe that a more in-depth and comprehensive study is warranted.

The aim of our research was to use representative samples of normal individuals to evaluate the relationship between serum albumin and pulmonary function based on NHANES samples (Ahluwalia et al., 2016). Our study provides a new method to assess pulmonary function and judge prognosis, a new strategy to prevent and treat respiratory failure, and a theoretical basis for the supplementation of albumin in patients with poor pulmonary function.

## 2 Materials and methods

### 2.1 Study population

**FIGURE 1:**
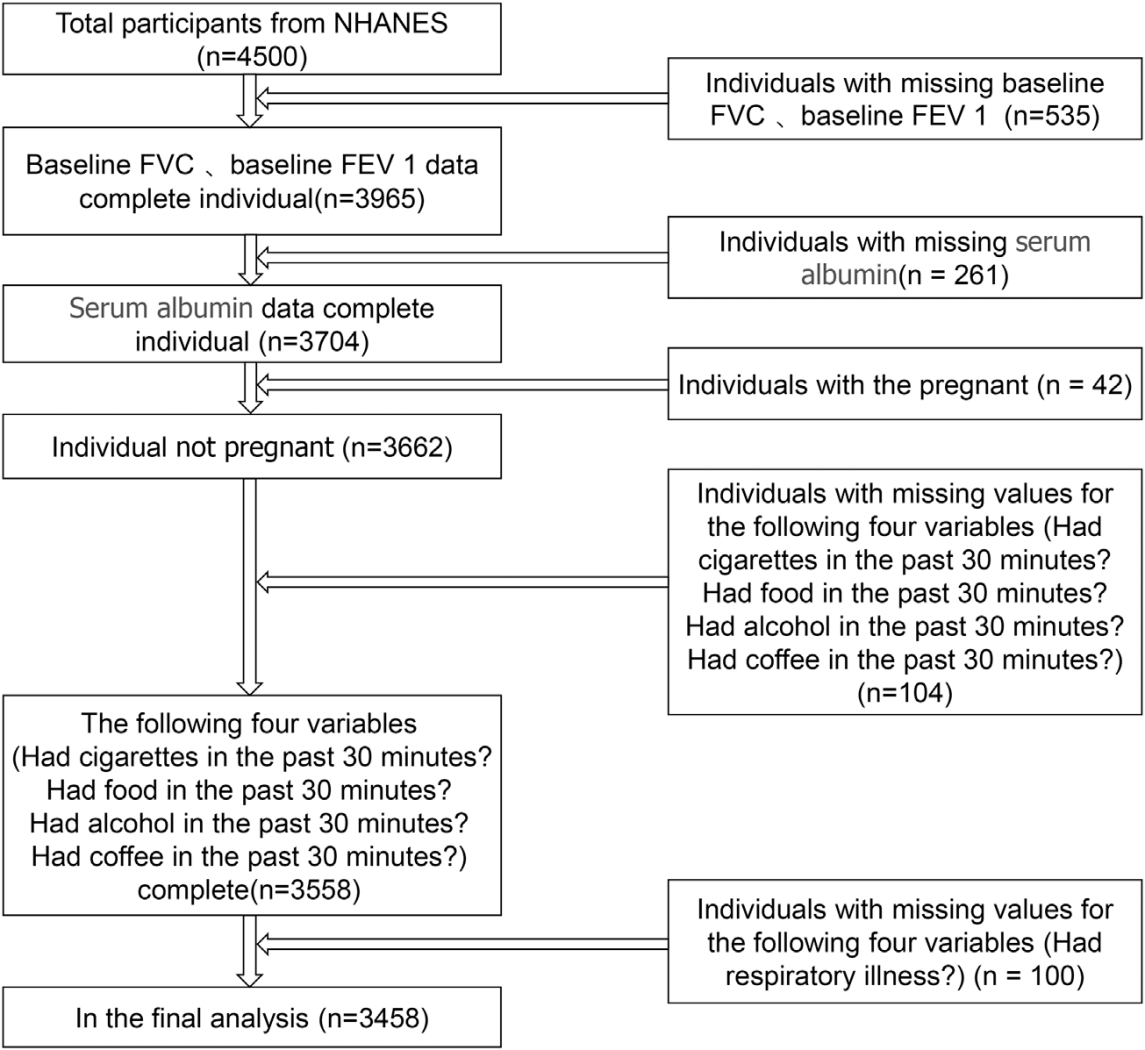
Participants’ screening flowchart.

The data were obtained from NHANES III, which is a representative investigation of the American civilian and non-institutionalized population conducted by the NCHS of the Centers for Disease Control and Prevention. Details of survey design and methods can be found at the NCHS website (http://www.cdc.gov/). In brief, an individual accepts an interview at home and is then invited to a mobile test center for further interviews, tests, and examinations. Our analysis is based on data recorded from 2013 to 2014, which represents a cycle of the NHANES database. The 2013–2014 database has the most recent data for FVC and FEV 1. The total number of participants exported from the NHANES database was 4,500. We excluded individuals with missing data on FVC, FEV 1, and serum albumin levels, and individuals whose data were collected 30 minutes after smoking, 30 minutes after eating, 30 minutes after drinking alcohol, or 30 minutes after drinking coffee. In addition, individuals who were pregnant or had missing data for respiratory illness history were also excluded. Ultimately, there were 3458 participants in our study (Figure 1).

### 2.2 Variables

Serum albumin (g/dl) was the exposure variable in this study. Certified laboratory specialists collected and processed blood samples at the mobile examination center, and then stored them in biorepositories. To maintain the integrity of the samples, certain methods and criteria were followed. As a bichromatic digital endpoint approach, the DcX800 method was used to detect the serum albumin concentration, in which serum albumin forms a complex with the Bromcresol Purple (BCP) reagent. The system then measures the change in absorbance at 600 nm. The content of albumin in the sample is directly proportional to the change in absorbance. We divided serum albumin levels into three groups according to low, medium, and high. The low group was ≥2.1–4.2 g/dl, the medium group was ≥4.2–4.5 g/dl, and the high group was ≥4.5 to ≤5.4 g/dl. These groupings were predetermined based on research literature that previously demonstrated an association between serum albumin and liver function or kidney function or respiratory function (Razeghi et al., 2012; Abbatecola et al., 2014; Labgaa et al., 2016; Hattori et al., 2018; Li et al., 2020). The outcome variables were FVC and FEV 1. We used the functional spirometry assessment procedure based on the latest standards of the American Thoracic Society (ATS). The prompts of the spirometry system were followed to begin the examination. The standardized patient (SP) took a deep breath and after the chart driver began to roll, the SP placed the tube in his or her mouth and began to blow out rapidly. The SP was encouraged to continue blowing for a minimum of 6 s, as required by the ATS. The SP was then asked to stop blowing and the mouthpiece was removed. The test was repeated as prompted by the spirometry system. The examination ended after five acceptable/repeatable tests were obtained. The following continuous covariates were included in our analysis: age, weight (kg), standing height (cm), systolic blood pressure (mmHg), serum glucose (mmol/L), cholesterol (mmol/L), alanine aminotransferase (ALT) (U/L), total calcium (mmol/L), sodium (mmol/L), potassium (mmol/L). In our analysis, the following categorical variables were included as covariates: Sex, race, cigarette smoking, education level, previous surgery, and respiratory disease. For more information about serum albumin, FVC, FEV 1, and covariates, please visit https://www.cdc.gov/nchs/nhanes/.

### 2.3 Statistical analysis

All analyses were performed using Empower (R) (www.empowerstats.com, X&Y solutions, inc. Boston, MA, United States) and R version 3.6.3 (http://www.R-project.org). Empower Stats is a statistical software based on the R language for data analysis. The software has powerful data processing functions, as well as comprehensive analysis functions. *p* < 0.05 was accepted to indicate statistical significance. To explain the considerable variance in the data set, we used a weighted variance estimation analysis. The relationship between the serum albumin level and FVC and FEV 1 was studied using a weighted multivariate logistic regression model. To compute the difference between groups, we used a weighted chi-squared test for the categorical data and a weighted linear regression model for the continuous variables. Subgroup analysis was carried out using hierarchical multiple regression analysis. The non-linear link between the serum albumin level and FVC and FEV 1 was addressed using smooth curve fitting and a generalized additive model. A recursive algorithm was used to compute the in-flection point of the association between the serum albumin level and FVC and FEV 1 when non-linearity was discovered. A piecewise linear regression model was used on both sides of the inflection point.

## 3 Results

### 3.1 Baseline characteristics of selected participants subsection

Our study included 3,458 participants (1,797 male and 1,661 female), and tertiles were used as a subcdivision of the weighted characteristics of the participants (Table 1). The results showed that as the albumin level increased, both FVC and FEV 1 showed a gradual upward trend in the low, medium, and high serum albumin groups, with a statistically significant difference (*p* < 0.001). Details of the statistically significant variables for the three groups (low, medium, and high albumin) are shown in Table 1, all with *p* < 0.05. There were no significant differences in diastolic blood pressure, creatinine, and cigarette smoking within the low, middle, and high tertiles of the serum albumin level (Table 1).

**TABLE 1:** Baseline characteristics of participants stratified by serum albumin tri-sectional quantiles.

Albumin (g/dl) Tertile	Low (≥2.1–4.2)	Middle (≥4.2–4.5)	High (≥4.5 to ≤5.4)	*p*-value
Globulin (g/dl)	3.07 ± 0.50	2.87 ± 0.40	2.75 ± 0.41	<0.001
Age, mean ± SD (years)	46.44 ± 13.55	44.37 ± 14.09	39.21 ± 14.29	<0.001
Weight (kg)	88.49 ± 24.64	81.10 ± 20.64	77.39 ± 17.99	<0.001
Standing Height (cm)	166.35 ± 9.21	167.93 ± 10.17	171.07 ± 9.79	<0.001
Systolic blood pressure (mmHg)	123.29 ± 18.01	120.91 ± 16.59	119.96 ± 15.03	<0.001
Diastolic blood pressure (mmHg)	72.40 ± 12.60	72.56 ± 11.84	72.31 ± 11.39	0.88
Glucose, serum (mmol/L)	5.98 ± 2.81	5.55 ± 1.91	5.21 ± 1.30	<0.001
Cholesterol (mmol/L)	4.89 ± 1.08	4.94 ± 1.02	5.07 ± 1.04	<0.001
Creatinine (umol/L)	77.40 ± 40.46	76.43 ± 22.10	78.69 ± 18.12	0.135
Alanine aminotransferase ALT (U/L)	23.97 ± 19.71	25.21 ± 19.61	26.95 ± 18.15	0.001
Total calcium (mmol/L)	2.30 ± 0.08	2.34 ± 0.07	2.39 ± 0.07	<0.001
Sodium (mmol/L)	138.72 ± 2.53	139.04 ± 2.13	139.04 ± 1.94	<0.001
Potassium (mmol/L)	3.92 ± 0.33	3.93 ± 0.33	3.89 ± 0.32	0.006
Albumin (g/dl)	3.94 ± 0.18	4.30 ± 0.08	4.66 ± 0.17	<0.001
Baseline FVC (ml)	3521.11 ± 912.79	3912.78 ± 1043.71	4374.11 ± 1061.16	<0.001
Baseline FEV 1 (ml)	2757.14 ± 739.08	3082.14 ± 843.10	3514.97 ± 887.13	<0.001
Gender (%)	—	—	—	<0.001
Male	33.7	51.6	68.4	—
Female	66.3	48.4	31.6	—
Race/Hispanic origin (%)	—	—	—	<0.001
Mexican American	9.3	11.9	11.2	—
Other Hispanic	10.5	10.5	9.2	—
Non-Hispanic white	30.2	33.7	39.8	—
Non-Hispanic black	38.5	25.9	17.1	—
Other races - Including multi-racial	11.6	17.9	22.6	—
Education level (%)	—	—	—	0.002
Less than 9th grade	6.5	7.1	6.3	—
9–11th grade	14.7	12.3	12	—
High school graduate	21.8	20	17.9	—
Some college or AA degree	33	33.3	31.1	—
College graduate or above	24	27.4	32.7	—
Thoracic/abdominal surgery (%)	—	—	—	<0.001
Yes	23.6	19.7	14.8	—
No	76.4	80.3	85.2	—
Respiratory disease (%)	—	—	—	<0.001
Yes	21.4	17.4	13.9	—
No	78.6	82.6	86.1	—
Cigarette (%)	—	—	—	0.516
Yes	2.9	2.3	2.2	—
No	97.1	97.7	97.8	—

Note: Continuous variables were presented as mean ± SD; Categorical variables were presented as n (%). FVC: forced vital capacity; FEV1: forced expiratory volume in one second.

### 3.2 Univariate analysis and stratified analysis of the association between serum albumin and pulmonary function

A positive association between the serum albumin level and pulmonary function was demonstrated by crude univariate analysis (Table 2). The reference group for each variable was the first group. For the baseline FVC analysis, compared with that the lower tertile group, the beta value (confidence interval (CI)) of the serum albumin level was 391.67 (308.23, 475.10) in the middle tertile group and 852.99 (768.11, 937.87) in the high tertile group, all at *p* < 0.0001. For the baseline FEV 1 analysis, compared with that in the lower tertile group, the beta value (CI) of the serum albumin level was 325 (256.68, 392.32) in the middle tertile group and 757.83 (688.33, 827.34) in the high tertile group, all at *p* < 0.0001. FVC and FEV 1 were also associated with globulin, age, gender, race, weight, education level, thoracic/abdominal surgery, respiratory disease, weight, standing height, systolic blood pressure, serum glucose, creatine, cholesterol, ALT, total calcium, sodium, potassium. In addition, smoking was associated with FVC, while it had no significant association with FEV 1. Diastolic blood pressure was not significantly associated with FVC and FEV 1. For further study, stratified analysis (Supplementary Table S1) was performed. In the stratified analysis, serum albumin levels and pulmonary function were positively correlated in almost all strata (Supplementary Table S1).

**TABLE 2:** Crude univariate analysis for Baseline FVC and Baseline FEV 1.

Exposure	Statistics	Baseline FVC (ml)	Baseline FEV 1 (ml)
		β(95%CI) *p*-value	β(95%CI) *p*-value
Albumin (g/dl)	4.32 ± 0.32	1147.13 (1043.99, 1250.27) <0.0001	1019.96 (935.63, 1104.29) <0.0001
Albumin (g/dl) Tertile			
Low	1027 (29.70%)	0	0
Middle	1261 (36.47%)	391.67 (308.23, 475.10) <0.0001	325.00 (256.68, 393.32) <0.0001
High	1170 (33.83%)	852.99 (768.11, 937.87) <0.0001	757.83 (688.33, 827.34) <0.0001
Globulin (g/dl)	2.89 ± 0.45	−676.11 (−751.30, -600.92) <0.0001	−474.29 (−537.20, −411.37) <0.0001
Globulin (g/dl) Tertile			
Low	1032 (29.90%)	0	0
Middle	1003 (29.06%)	−316.83 (−406.23, −227.42) <0.0001	−239.90 (−314.57, −165.24) <0.0001
High	1417 (41.05%)	−691.81 (−774.32, −609.30) <0.0001	−491.37 (−560.28, −422.45) <0.0001
Age (years)	43.23 ± 14.31	−27.57 (−29.88, −25.26) <0.0001	−30.84 (−32.62, −29.06) <0.0001
Age (years) Tertile			
Low	1136 (32.85%)	0	0
Middle	1114 (32.22%)	−306.62 (−389.10, −224.15) <0.0001	−409.91 (−474.04, −345.77) <0.0001
High	1208 (34.93%)	−909.28 (−990.12, −828.44) <0.0001	−1016.22 (−1079.09, −953.36) <0.0001
Gender			
Male	1797 (51.97%)	0	0
Female	1661 (48.03%)	−1338.35 (−1393.93, −1282.77) <0.0001	−986.46 (−1035.34, −937.58) <0.0001
Race/Hispanic origin			
Mexican American	376 (10.87%)	0	0
Other Hispanic	349 (10.09%)	−249.96 (−397.60, −102.33) 0.0009	−185.12 (−309.50, −60.73) 0.0036
Non-Hispanic white	1201 (34.73%)	326.93 (209.56, 444.31) <0.0001	148.98 (50.09, 247.87) 0.0032
Non-Hispanic black	922 (26.66%)	−492.46 (−614.00, −370.93) <0.0001	−415.34 (−517.74, −312.94) <0.0001
Other races (a)	610 (17.64%)	−307.81 (−438.03, −177.58) <0.0001	−200.60 (−310.32, −90.87) 0.0003
Education level			
Less than 9th grade	230 (6.65%)	0	0
9-11th grade (b)	446 (12.90%)	209.87 (40.55, 379.19) 0.0152	191.22 (51.48, 330.96) 0.0074
High school graduate (c)	685 (19.81%)	266.25 (107.30, 425.21) 0.0010	239.80 (108.62, 370.99) 0.0003
Some college or AA degree	1123 (32.48%)	308.87 (157.91, 459.84) <0.0001	302.93 (178.35, 427.52) <0.0001
College graduate or above	974 (28.17%)	398.40 (245.49, 551.31) <0.0001	373.79 (247.59, 499.99) <0.0001
Thoracic/abdominal surgery			
Yes	664 (19.20%)	0	0
No	2794 (80.80%)	445.05 (355.87, 534.23) <0.0001	427.17 (353.82, 500.53) <0.0001
Respiratory disease			
Yes	602 (17.41%)	0	0
No	2856 (82.59%)	153.79 (60.02, 247.56) 0.0013	153.01 (75.55, 230.46) 0.0001
Cigarette			
Yes	85 (2.46%)	0	0
No	3373 (97.54%)	−315.96 (−545.70, −86.23) 0.0071	−177.43 (−367.42, 12.57) 0.0673
Weight (kg)	82.02 ± 21.55	10.50 (8.88, 12.11) <0.0001	7.23 (5.89, 8.58) <0.0001
Weight (kg) Tertile			
Low	1144 (33.25%)	0	0
Middle	1146 (33.30%)	465.60 (380.63, 550.57) <0.0001	309.38 (238.47, 380.29) <0.0001
High	1151 (33.45%)	597.59 (512.72, 682.47) <0.0001	412.53 (341.70, 483.35) <0.0001
Standing Height (cm)	168.53 ± 9.95	78.58 (76.14, 81.02) <0.0001	58.59 (56.36, 60.81) <0.0001
Standing Height (cm) Tertile			
Low	1137 (33.03%)	0	0
Middle	1153 (33.50%)	785.84 (721.66, 850.01) <0.0001	572.95 (515.93, 629.98) <0.0001
High	1152 (33.47%)	1776.28 (1712.09, 1840.47) <0.0001	1327.59 (1270.55, 1384.63) <0.0001
Systolic blood pressure (mmHg)	121.30 ± 16.57	−7.56 (−9.73, −5.38) <0.0001	−8.76 (−10.55, −6.98) <0.0001
Systolic blood pressure (mmHg) Tertile			
Low	1052 (31.72%)	0	0
Middle	1105 (33.31%)	214.05 (124.85, 303.25) <0.0001	124.44 (50.98, 197.89) 0.0009
High	1160 (34.97%)	−137.99 (−226.15, −49.83) 0.0022	−230.19 (−302.79, −157.59) <0.0001
Diastolic blood pressure (mmHg)	72.43 ± 11.92	3.49 (0.45, 6.53) 0.0246	0.39 (−2.13, 2.91) 0.7615
Diastolic blood pressure (mmHg) Tertile			
Low	991 (29.88%)	0	0
Middle	1211 (36.51%)	65.45 (−24.05, 154.95) 0.1519	4.69 (−69.41, 78.80) 0.9012
High	1115 (33.61%)	81.92 (−9.30, 173.13) 0.0785	−19.25 (−94.78, 56.27) 0.6173
Glucose, serum (mmol/L)	5.56 ± 2.08	−60.94 (−77.92, −43.97) <0.0001	−58.43 (−72.42, −44.44) <0.0001
Glucose, serum (mmol/L) Tertile			
Low	1114 (32.22%)	0	0
Middle	1121 (32.42%)	−36.03 (−124.04, 51.98) 0.4223	−46.60 (−118.91, 25.72) 0.2067
High	1223 (35.37%)	−274.08 (−360.24, −187.92) <0.0001	−311.76 (−382.56, −240.96) <0.0001
Cholesterol (mmol/L)	4.97 ± 1.05	−107.85 (−141.69, −74.01) <0.0001	−112.11 (−139.99, −84.23) <0.0001
Cholesterol (mmol/L) Tertile			
Low	1132 (32.75%)	0	0
Middle	1163 (33.64%)	−46.14 (−133.22, 40.94) 0.2991	−58.36 (−130.12, 13.40) 0.1111
High	1162 (33.61%)	−227.48 (−314.58, −140.38) <0.0001	−248.16 (−319.94, −176.38) <0.0001
Creatinine (umol/L)	77.48 ± 27.85	6.47 (5.21, 7.73) <0.0001	4.38 (3.33, 5.42) <0.0001
Creatinine (umol/L) Tertile			
Low	1121 (32.42%)	0	0
Middle	1164 (33.66%)	603.86 (521.24, 686.48) <0.0001	434.63 (365.27, 503.98) <0.0001
High	1173 (33.92%)	853.51 (771.05, 935.98) <0.0001	609.64 (540.42, 678.87) <0.0001
Alanine aminotransferase ALT (U/L)	25.43 ± 19.19	7.29 (5.45, 9.13) <0.0001	5.05 (3.53, 6.58) <0.0001
Alanine aminotransferase ALT (U/L) Tertile			
Low	1140 (32.99%)	0	0
Middle	1114 (32.23%)	272.72 (186.36, 359.07) <0.0001	169.66 (97.86, 241.46) <0.0001
High	1202 (34.78%)	530.45 (445.71, 615.20) <0.0001	374.95 (304.49, 445.40) <0.0001
Total calcium (mmol/L) Tertile			
Low	812 (23.49%)	0	0
Middle	1231 (35.61%)	109.40 (14.98, 203.81) 0.0232	119.62 (41.71, 197.54) 0.0026
High	1414 (40.90%)	209.10 (117.14, 301.05) <0.0001	217.11 (141.23, 292.99) <0.0001
Sodium (mmol/L)	138.95 ± 2.20	−4.52 (−20.71, 11.67) 0.5844	−3.53 (−16.92, 9.85) 0.6048
Sodium (mmol/L) Tertile			
Low	721 (20.86%)	0	0
Middle	1358 (39.28%)	83.08 (−13.37, 179.53) 0.0914	110.82 (31.19, 190.46) 0.0064
High	1378 (39.86%)	0.98 (−95.22, 97.18) 0.9841	5.43 (−74.01, 84.86) 0.8935
Potassium (mmol/L)	3.91 ± 0.33	159.56 (50.72, 268.41) 0.0041	48.95 (−41.11, 139.00) 0.2868
Potassium (mmol/L) Tertile			
Low	1023 (29.59%)	0	0
Middle	944 (27.31%)	160.12 (65.80, 254.43) 0.0009	118.45 (40.42, 196.49) 0.0029
High	1490 (43.10%)	155.63 (70.78, 240.48) 0.0003	65.50 (−4.71, 135.71) 0.0675

Note: Continuous variables were presented as mean ± SD; Categorical variables were presented as n (%); The first group was used as the reference (β = 0) for each univariate analysis group; (a) Including Multi-Racial; (b)Includes 12th grade with no diploma; (c) GED, or equivalent. Weighted by: Full sample mobile examination center exam weight. Abbreviations: FVC: forced vital capacity; FEV1, Forced expiratory volume in one second. The first group was used as the reference for each univariate analysis group.

### 3.3 Multiple regression equation analysis of the association between serum albumin and pulmonary function

Similarly, multivariate analysis revealed a positive relationship between serum albumin and pulmonary function (Table 3). In different models, the beta values of both FVC and FEV 1 increased approximately with increasing albumin levels. In the unadjusted model, compared with that in the lower tertile group, higher albumin levels were associated with higher FVC (β = 852.99, 95% CI = 768.11–937.87, *p* < 0.0001) and FEV 1 (β = 325.00, 95% CI = 256.68–393.32, *p* < 0.0001). In the model I which adjusted for gender, high albumin levels were also associated with higher FVC (β = 421.00, 95% CI = 349.7–492.2, *p* < 0.05) and FEV 1 (β = 450.10, 95% CI = 388.10–512.20, *p* < 0.001). In the fully adjusted model IV, high albumin levels were associated with higher FVC (β = 63.61, 95% CI = 1.99–125.23, *p* < 0.05) and FEV 1 (β = 119.90, 95% CI = 65.77–174.02, *p* < 0.0001). The rough model was unadjusted. The covariates used for adjustment in models are detailed in Table 3.

**TABLE 3:** Relationship between serum and serum albumin and Pulmonary Function (multiple regression equation analysis).

Outcome	Rough model	Model I	Model II	Model III	Model IV
	β (95%CI) *p*-value	β (95%CI) *p*-value	β (95%CI) *p*-value	β (95%CI) *p*-value	β (95%CI) *p*-value
Y= Baseline FVC (ml)					
Albumin (g/dl)	1147.13 (1043.99, 1250.27) <0.0001	581.2 (493.4, 669.0) <0.001	314.1 (232.5, 395.6) <0.001	208.2 (132.3, 284.0) <0.001	103.74 (20.34, 187.14) 0.0148
Albumin (g/dl) Tertile					
Low	0	0	0	0	0
Middle	391.67 (308.23, 475.10) <0.0001	168.3 (100.2, 236.4) <0.001	111.0 (49.5, 172.5) <0.001	78.1 (21.8, 134.4) 0.007	49.10 (−1.31, 99.51) 0.0563
High	852.99 (768.11, 937.87) <0.0001	421.0 (349.7, 492.2) <0.001	228.5 (162.9, 294.1) <0.001	157.3 (96.6, 218.1) <0.001	63.61 (1.99, 125.23) 0.0431
Y= Baseline FEV 1 (ml)					
Albumin (g/dl)	1019.96 (935.63, 1104.29) <0.0001	620.0 (543.7, 696.3) <0.001	315.2 (250.3, 380.0) <0.001	245.7 (182.9, 308.5) <0.001	191.66 (118.47, 264.85) <0.0001
Albumin (g/dl) Tertile					
Low	0	0	0	0	0
Middle	325.00 (256.68, 393.32) <0.0001	165.9 (106.6, 225.2) <0.001	100.7 (51.8, 149.7) <0.001	76.9 (30.2, 123.5) 0.001	61.32 (17.05, 105.60) 0.0067
High	757.83 (688.33, 827.34) <0.0001	450.1 (388.1, 512.2) <0.001	231.1 (178.9, 283.3) <0.001	183.1 (132.7, 233.4) <0.001	119.90 (65.77, 174.02) <0.0001

Note: Abbreviations: FVC: forced vital capacity; FEV1: Forced expiratory volume in one second. Weighted by: Full sample mobile examination center exam weight. Outcome variable: baseline FVC; Baseline FEV, 1. Exposure variable: serum albumin (mg/dl). Rough model: variables unadjusted. Model I adjusted by gender; Model II, adjusted by: gender, age; Model II, adjusted by: gender, age, race; Model IV, adjusted by: globulin, age, gender, race/hispanic origin, education level, thoracic/abdominal surgery (yes, no), respiratory disease (yes, no), cigarette (yes, no), weight, standing height, systolic blood pressure, diastolic blood pressure, glucose, serum, cholesterol, creatinine, alanine aminotransferase alt, total calcium, sodium, potassium.

### 3.4 Smooth curve fitting, threshold effect and saturation effect analyses between serum albumin levels and pulmonary function

**FIGURE 2:**
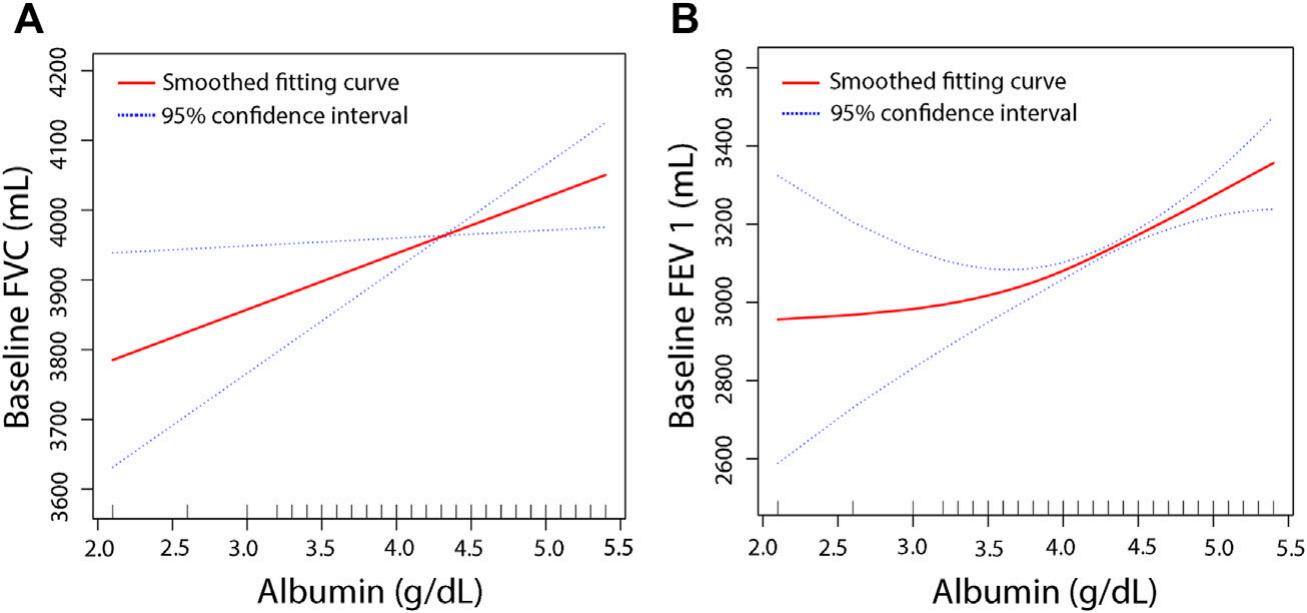
The association between serum albumin and pulmonary function. The smooth curve fit between the variables is indicated by the red line. The fit’s 95 percent confidence interval is represented by the blue bar. Weighted by: Full sample mobile examination center exam weight. Adjusted for, globulin (smooth), age (smooth), gender, education level, race, surgery (yes, no), respiratory disease (yes, no), cigarette (yes, no), weight (smooth), standing height (smooth), diastolic blood pressure (smooth), systolic blood pressure (smooth), glucose, serum (smooth), cholesterol (smooth), creatinine (smooth), ALT (smooth), total calcium (smooth), sodium (smooth), potassium (smooth). **(A)** Solid line plot of curve fit whose main variables are baseline albumin and FVC. **(B)** Solid line plot of curve fit whose main variables are baseline albumin and FEV 1.

To further clarify the relationship between the serum albumin level and pulmonary function, we performed smooth curve fitting (Figure 2) between serum albumin levels and pulmonary function. To identify the serum albumin level at which the baseline FVC and FEV 1 start to increase, threshold effect and saturation effect analyses were per-formed (Table 4). The adjusted smoothed curve fit plot showed a linear relationship between serum albumin levels and FVC: for every 1 g/dl increase in serum albumin level, FVC increased by 80.40 ml (95% CI = 11.18–149.61, *p* = 0.0229) (Figure 2A,B, Table 4). In addition, serum albumin and FEV 1 showed a non-linear relationship. When the serum albumin level reached the inflection point (3.8 g/dl), FEV 1 increased with in-creasing serum albumin and the correlation coefficient β was 205.55 (95% CI = 140.15–270.95, *p* < 0.0001). When serum albumin was less than 3.8 g/dl, there was no significant relationship between serum albumin and FVC (*p* = 0.3661) (Table 4). The covariates used for adjustment are detailed in Table 4.

**TABLE 4:** Analysis of threshold effect and saturation effect.

Outcome	Baseline FVC (ml)	Baseline FEV 1 (ml)
	β (95%CI) *p*-value	β (95%CI) *p*-value
Model I		
A straight-line effect	80.40 (11.18, 149.61) 0.0229	178.60 (117.92, 239.27) <0.0001
Model II		
Fold points (K)	4.5	3.8
< K-segment effect 1	100.26 (8.69, 191.83) 0.0320	−133.94 (−424.38, 156.49) 0.3661
>K-segment Effect 2	29.65 (−138.44, 197.74) 0.7296	205.55 (140.15, 270.95) <0.0001
Effect size difference of 2 *versus* 1	−70.61 (−283.74, 142.52) 0.5161	339.50 (30.96, 648.03) 0.0311
Equation predicted values at break points	4140.47 (4083.30, 4197.63)	2571.94 (2513.61, 2630.28)
Log likelihood ratio tests	0.515	0.03

Note: Abbreviations: CI, confidence interval; FVC: forced vital capacity; FEV1: Forced expiratory volume in one second. Weighted by: Full sample mobile examination center exam weight. Outcome variable: AAC, total 24 score. Exposure variable: serum uric acid (mg/dl). Adjusted for age, gender, race/hispanic origin, education level, thoracic/abdominal surgery, respiratory disease, cigarette, weight, standing height, systolic blood pressure, diastolic blood pressure, glucose, serum, cholesterol, creatinine, alanine aminotransferase alt, globulin. β represents the slope of the curve, β for segments with p < 0.05 was statistically significant. The K value is the inflection point value, which is the level of serum albumin content at which the relationship between serum albumin and lung function changes.

### 3.5 Smooth curve fitting for stratification, threshold effect and saturation effect analyses for stratification

**FIGURE 3:**
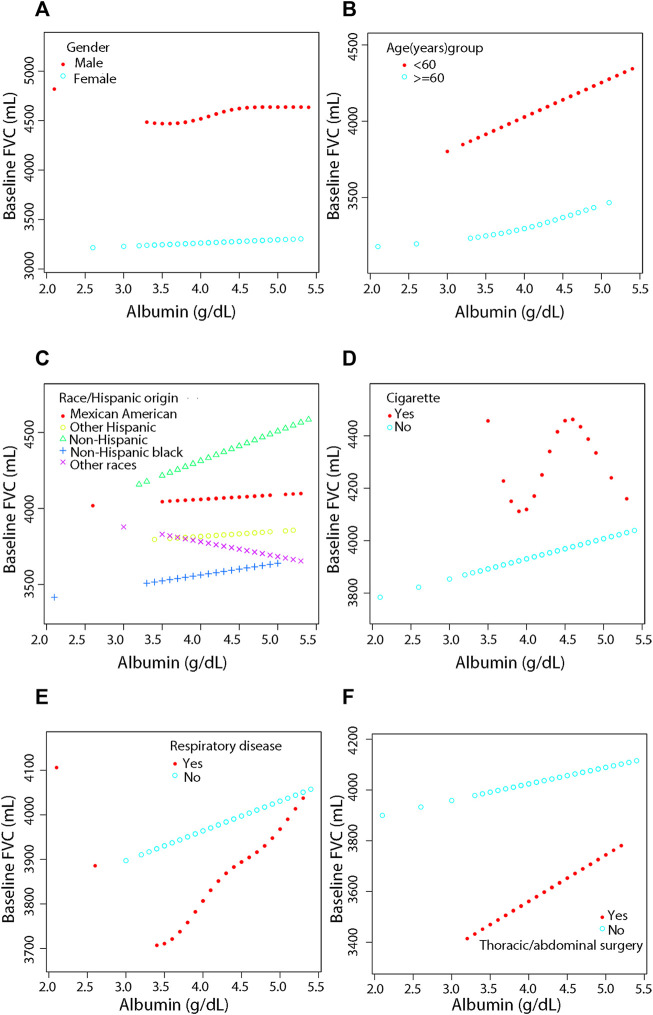
Association between serum albumin and Pulmonary Function (FVC) by covariate variables (gender, age, race, cigarette, respiratory disease, surgery). Weighted by: Exam weight in a full sample mobile examination center. Adjusted for globulin (smooth), age (smooth), gender, race, education level, surgery (yes, no), respiratory disease (yes, no), cigarette (yes, no), weight (smooth), standing height (smooth), diastolic blood pressure (smooth), systolic blood pressure (smooth), glucose, serum (smooth), cholesterol (smooth), creatinine (smooth), ALT (smooth), total calcium (smooth), sodium (smooth), potassium (smooth). **(A)** Stratified by gender. **(B)** Stratified by age dichotomy. **(C)** Stratified race. **(D)** Stratified by cigarette. **(E)** Stratified by respiratory disease. **(F)** Stratified by surgery.

**FIGURE 4:**
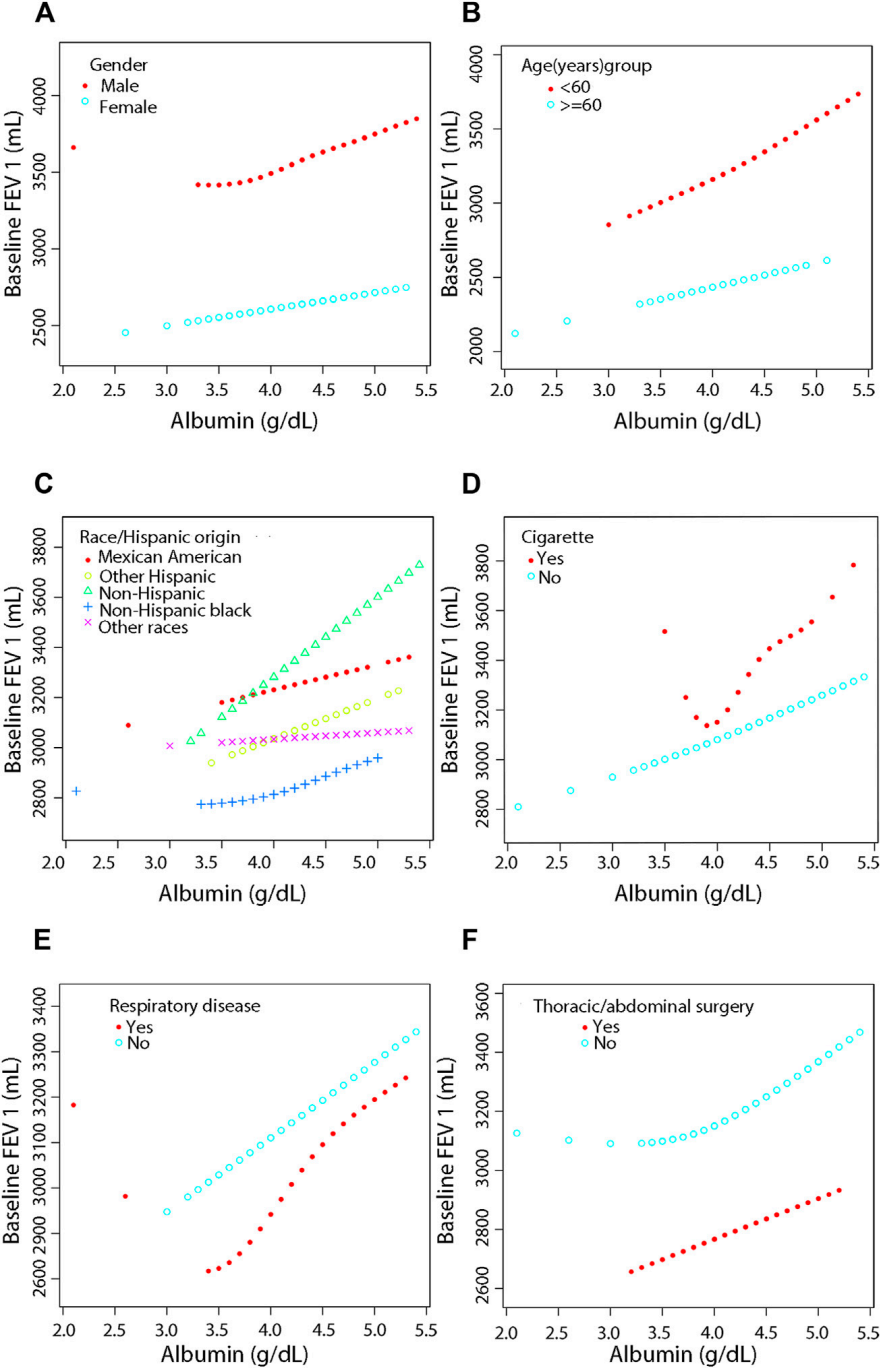
Association between serum albumin and Pulmonary Function (FEV 1) by covariate variables (gender, age, race, cigarette, respiratory disease, surgery). Weighted by: Exam weight in a full sample mobile examination center. Adjusted for globulin (smooth), age (smooth), gender, education level, race, surgery (yes, no), respiratory disease (yes, no), cigarette (yes, no), weight (smooth), standing height (smooth), diastolic blood pressure (smooth), systolic blood pressure (smooth), glucose, serum (smooth), cholesterol (smooth), creatinine (smooth), ALT (smooth), total calcium (smooth), sodium (smooth), potassium (smooth). **(A)** Stratified by gender. **(B)** Stratified by age dichotomy. **(C)** Stratified race. **(D)** Stratified by cigarette. **(E)** Stratified by respiratory disease. **(F)** Stratified by surgery.

For more detailed analysis, smooth fitting curves were drawn for different strata of the six covariates (Figure 3, Figure 4). Threshold effect and saturation effect analysis were performed to clarify the changes in baseline FEV and FEV 1 with increase of serum albumin in the different strata of each covariate (Supplementary Table S3–S13) respectively. The K value in tables is the inflection point value, which is the level of serum albumin content at which the relationship between serum albumin and lung function changes. There is no K value when the relationship between serum albumin and the outcome variable shows a straight-line effect.

After adjustment, the results showed a linear positive correlation between serum albumin and both FVC (β = 236.20, 95% CI = 156.51–315.90, *p* < 0.0001) (Figure 3B) and FEV 1 (β = 375.12, 95% CI = 301.32–448.92, *p* < 0.0001) (Figure 4B) in those aged less than 60 years (Supplementary Table S3). In the stratification of race, serum albumin and FVC were linearly and positively (β = 127.80, 95% CI = 0.84–254.75, *p* = 0.0488) correlated in the Non-Hispanic black population (Supplementary Table S4, Figure 3C). In the Mexican American population (β = 174.48, 95% CI = 21.52–327.44, *p* = 0.0260), Non-Hispanic white population (β = 266.81, 95% CI = 151.56–382.05, *p* < 0.0001), and Non-Hispanic black population (β = 155.27, 95% CI = 38.09–272.45, *p* = 0.0096), serum albumin was linearly and positively correlated with FEV 1 in all three populations and the correlations were significantly different (P-interaction = 0.001) (Supplementary Table S4, Figure 4C).

In the smoking population, serum albumin and FEV 1 showed a segmental effect, and when serum albumin was <3.8 g/dl, serum albumin and FEV 1 were negatively correlated (β = -3577.38, 95% CI = −6506.52 to −648.24, *p* = 0.0199) (Supplementary Table S5, Figure 4D). In addition, serum albumin was linearly and positively correlated with both FVC (β = 80.95, 95% CI = 11.11–150.79, *p* = 0.0232) and FEV 1 (β = 178.72, 95% CI = 117.44–239.99, *p* < 0.0001) in the nonsmoking population (Supplementary Table S5, Figure 3D and Figure 4D).

Serum albumin was linearly and positively correlated with FEV 1 in those with previous respiratory disease (β = 228.90, 95% CI = 73.89–383.91, *p* = 0.0040). Serum albumin was also linearly and positively correlated with FEV 1 in those who had not had respiratory disease (β = 163.86, 95% CI = 98.00–229.73, *p* < 0.0001), and the correlation was similar in both groups (P-interaction = 0.256) (Supplementary Table S6, Figure 4E). In those who had thoracic or abdominal surgery, serum albumin showed a linear positive correlation with FEV 1 with a correlation coefficient of 160.38 (95% CI = 28.63–292.13, *p* = 0.0173). In those who did not have thoracic or abdominal surgery, serum albumin showed a segmental effect with FEV 1, with serum albumin levels increasing with FEV 1 when serum albumin levels were >3.8 g/dl with a correlation coefficient of 209.36 (95% CI = 136.42–282.30, *p* < 0.0001). The covariates used for adjustment are detailed in Supplementary Table S2–S7.

## 4 Discussion

Worldwide, respiratory diseases are one of the leading causes of mortality and represent a serious health threat (Wang et al., 2013; Heron, 2019; Gayle et al., 2005-2015; Siegel et al., 2022; Koskela et al., 2005; Faustini et al., 2013). The many components of pulmonary function provide a valuable tool to assess respiratory system health and disorders in clinical settings (Liang et al., 2012). However, pulmonary function tests are contraindicated in pulmonary maculopathy, severe chest trauma, in the ICU, within 1 month of myocardial infarction, and for 3 weeks after thoracic surgery; therefore, the currently used pulmonary function tests are sometimes insufficient to support the decision to provide necessary respiratory support (Cooper, 2011; Altree et al., 2019; Rivero-Yeverino, 2019).

Many previous studies have explored alternatives to pulmonary function tests. We reviewed a large amount of data and literature, which suggested that serum albumin might a good indicator. We then analyzed the relationship between the serum albumin level and pulmonary function based on the NHANES database, which is a rigorous and comprehensive database. Many scholars have used this database to obtain meaningful results. Chen et al. used the NHANES database to analyze the relationship between weight change and mortality in Americans across adulthood (Chen et al., 2019). They found that stable obesity in adulthood, weight gain from young adulthood to midlife, and weight loss from midlife to late adulthood were associated with an increased risk of death. Based on Big Data, more unknown disease risk factors can be discovered, more accurate diagnosis and prognosis predictions become possible, and further innovative disease solutions can be proposed (Big hopes for big data, 2020). Large amounts valuable data were obtained from this database for analysis.

Serum albumin is related to cerebral ischemia (Turhan et al., 2006), Alzheimer’s disease (Skillbäck et al., 2017; Bode et al., 2018; Kim et al., 2020), Crohn’s disease (Su et al., 2019), liver cirrhosis (Chen et al., 2011; Oettl et al., 2013; Arroyo et al., 2014), chronic heart failure (Jabbour et al., 2014), acute kidney injury (Bang et al., 2018; Thongprayoon et al., 2018; Lv et al., 2021), chronic kidney disease (Stoycheff et al., 2009; Zhang et al., 2015) and other diseases; however, the relationship between serum albumin and pulmonary function is unclear. Several studies have shown that in some acute respiratory diseases that lead to reduced pulmonary function, such as acute respiratory distress and acute lung, serum albumin increases capillary permeability, causing it to leak from blood vessels into tissue fluid or to escape into the alveolar space, resulting in reduced serum albumin levels (Fanali et al., 2012). Besides, a reduced serum albumin level is a recognized clinical sign of malnutrition, and malnutrition causes respiratory muscle weakness and reduces pulmonary function (Kuzuya et al., 2007; Itoh et al., 2013; Rozga et al., 2013; Zhang et al., 2017; Arigliani et al., 2018; Eckart et al., 2020; Ferdous et al., 2021). Therefore, serum albumin can be increased to prevent respiratory failure. The results of the present study indicated that serum albumin correlated positively with important indicators of pulmonary function, which corroborates the above point.

Asl et al. carried out a cross-sectional study of 35 patients with COPD and cachexia found that serum albumin correlated positively with pulmonary function (Görek Dilektaşli et al., 2009), which agrees with our results. That study was the first cross-sectional study to include the association between albumin and pulmonary function; however, the study population was limited and the sample size was small. Compared with that study, our study has a higher sample size, a broader study population, and a more statistically rigorous approach by removing the effect of confounding factors.

The association of serum albumin with liver and kidney function has been widely demonstrated. In the rough endoplasmic reticulum, hepatocytes manufacture serum albumin in circular polysomes. Therefore, serum albumin levels will drop as liver function is impaired (Spinella et al., 2016). In contrast, there is a direct loss of albumin during nephrotic syndrome, peritoneal dialysis, and high-flux hemodialysis (Mehrotra et al., 2011). Whether the intrinsic mechanism of the effect of serum albumin on pulmonary function is achieved by affecting liver and kidney function should be the subject of further clinical and basic research. However, this study provides a guide for predicting pulmonary function and predicting respiratory failure in specific populations.

There are still certain limits to our research. First, although the sample size was relatively large, increasing the number of subjects would make the research results more convincing. Second, the cross-sectional study itself has certain limitations. For example, it cannot explain the causal relationship and can only indicate the correlation between two factors on a cross-sectional basis. Moreover, although we controlled for related confounding factors, interference by other confounding factors cannot be ruled out, thus further large-sample prospective studies and more refined data are needed for confirmation.

## 5 Conclusion

The apparent positive correlation between serum albumin levels and pulmonary function provides a credible complement to pulmonary function monitoring in specific populations, and serum albumin might become a promising indicator of when to administer respiratory support therapy in the future. Our results also provide the theoretical basis and data support for improving pulmonary function in critically ill patients *via* albumin administration. Appropriate supplementation with albumin might provide a ventilator-free treatment for those with critical pulmonary function values.

## Data Availability

The publicly available datasets used in this study can be found on the Centers for Disease Control and Prevention (CDC) website at: https://www.cdc.gov/nchs/nhanes/index.htm.
