# Supplementary material for: Supplementation of serum albumin is associated with improved pulmonary function: NHANES 2013–2014
Source: Front Physiol. 2022 Oct 3;13:948370. doi: 10.3389/fphys.2022.948370 (PMC9574070; doi:10.3389/fphys.2022.948370)
Supplement: Supplementary file 1 [file Table1.DOCX]

**Table S1. Stratified analysis between serum albumin and baseline FVC, serum albumin and baseline FEV 1.**

| **Sub-group**  **X= Albumin (g/dL)** **Tertile** | **N** | **Baseline FVC (mL)**  **β (95%CI) P value** | **Baseline FEV 1 (mL)**  **β (95%CI) P value** |
| --- | --- | --- | --- |
| Globulin (g/dL) Tertile |  |  |  |
| Low |  |  |  |
| Low | 186 | 0 | 0 |
| Middle | 373 | 286.64 (99.21, 474.08) 0.0028 | 276.26 (119.57, 432.94) 0.0006 |
| High | 473 | 744.30 (563.58, 925.02) <0.0001 | 729.84 (578.77, 880.91) <0.0001 |
| Middle |  |  |  |
| Low | 247 | 0 | 0 |
| Middle | 386 | 303.25 (146.42, 460.09) 0.0002 | 282.33 (153.22, 411.45) <0.0001 |
| High | 370 | 676.62 (518.47, 834.78) <0.0001 | 647.01 (516.81, 777.21) <0.0001 |
| High |  |  |  |
| Low | 592 | 0 | 0 |
| Middle | 500 | 340.58 (228.73, 452.42) <0.0001 | 285.07 (193.53, 376.61) <0.0001 |
| High | 325 | 718.61 (591.48, 845.73) <0.0001 | 649.87 (545.82, 753.92) <0.0001 |
| Age (years) Tertile |  |  |  |
| Low |  |  |  |
| Low | 227 | 0 | 0 |
| Middle | 371 | 474.76 (316.95, 632.57) <0.0001 | 387.72 (261.50, 513.95) <0.0001 |
| High | 538 | 887.58 (739.36, 1035.80) <0.0001 | 762.11 (643.55, 880.67) <0.0001 |
| Middle |  |  |  |
| Low | 362 | 0 | 0 |
| Middle | 421 | 430.90 (297.66, 564.13) <0.0001 | 349.36 (246.34, 452.38) <0.0001 |
| High | 331 | 874.44 (733.07, 1015.80) <0.0001 | 696.54 (587.24, 805.85) <0.0001 |
| High |  |  |  |
| Low | 438 | 0 | 0 |
| Middle | 469 | 207.97 (82.63, 333.30) 0.0012 | 143.34 (49.77, 236.90) 0.0027 |
| High | 301 | 389.43 (248.21, 530.66) <0.0001 | 311.51 (206.09, 416.94) <0.0001 |
| Gender |  |  |  |
| Male |  |  |  |
| Low | 346 | 0 | 0 |
| Middle | 651 | 354.40 (235.27, 473.53) <0.0001 | 346.72 (243.01, 450.44) <0.0001 |
| High | 800 | 616.98 (501.76, 732.19) <0.0001 | 654.66 (554.35, 754.96) <0.0001 |
| Female |  |  |  |
| Low | 681 | 0 | 0 |
| Middle | 610 | 56.13 (-19.36, 131.61) 0.1452 | 60.44 (-4.97, 125.86) 0.0703 |
| High | 370 | 253.03 (165.58, 340.49) <0.0001 | 266.84 (191.05, 342.62) <0.0001 |
| Race/Hispanic origin |  |  |  |
| Mexican American |  |  |  |
| Low | 95 | 0 | 0 |
| Middle | 150 | 359.68 (127.66, 591.71) 0.0025 | 270.81 (86.45, 455.17) 0.0042 |
| High | 131 | 659.64 (421.17, 898.10) <0.0001 | 579.55 (390.07, 769.02) <0.0001 |
| Other Hispanic |  |  |  |
| Low | 108 | 0 | 0 |
| Middle | 133 | 368.86 (125.02, 612.70) 0.0032 | 365.67 (160.21, 571.14) 0.0005 |
| High | 108 | 658.59 (402.42, 914.77) <0.0001 | 616.32 (400.46, 832.19) <0.0001 |
| Non-Hispanic white |  |  |  |
| Low | 310 | 0 | 0 |
| Middle | 425 | 298.72 (147.44, 450.01) 0.0001 | 255.23 (128.26, 382.19) <0.0001 |
| High | 466 | 888.68 (740.23, 1037.13) <0.0001 | 825.20 (700.61, 949.79) <0.0001 |
| Non-Hispanic black |  |  |  |
| Low | 395 | 0 | 0 |
| Middle | 327 | 407.63 (276.53, 538.73) <0.0001 | 321.84 (210.11, 433.57) <0.0001 |
| High | 200 | 844.59 (692.41, 996.76) <0.0001 | 711.69 (582.00, 841.38) <0.0001 |
| Other races - Including multi-racial |  |  |  |
| Low | 119 | 0 | 0 |
| Middle | 226 | 325.62 (110.19, 541.06) 0.0032 | 285.44 (108.39, 462.48) 0.0017 |
| High | 265 | 581.12 (371.22, 791.02) <0.0001 | 549.90 (377.41, 722.39) <0.0001 |
| Education level |  |  |  |
| Less than 9th grade |  |  |  |
| Low | 67 | 0 | 0 |
| Middle | 89 | 363.42 (57.19, 669.65) 0.0209 | 286.28 (44.51, 528.05) 0.0212 |
| High | 74 | 400.43 (81.15, 719.71) 0.0147 | 351.80 (99.72, 603.87) 0.0067 |
| 9-11th grade |  |  |  |
| Low | 151 | 0 | 0 |
| Middle | 155 | 308.50 (87.46, 529.54) 0.0065 | 206.55 (24.26, 388.84) 0.0269 |
| High | 140 | 805.02 (578.21, 1031.83) <0.0001 | 699.66 (512.62, 886.71) <0.0001 |
| High school graduate |  |  |  |
| Low | 224 | 0 | 0 |
| Middle | 252 | 414.08 (235.67, 592.49) <0.0001 | 356.75 (205.78, 507.73) <0.0001 |
| High | 209 | 944.70 (757.85, 1131.54) <0.0001 | 866.59 (708.48, 1024.71) <0.0001 |
| Some college or AA degree |  |  |  |
| Low | 339 | 0 | 0 |
| Middle | 420 | 353.55 (209.19, 497.92) <0.0001 | 308.77 (190.22, 427.32) <0.0001 |
| High | 364 | 916.82 (767.58, 1066.06) <0.0001 | 816.75 (694.20, 939.31) <0.0001 |
| College graduate or above |  |  |  |
| Low | 246 | 0 | 0 |
| Middle | 345 | 459.31 (289.11, 629.51) <0.0001 | 376.09 (240.57, 511.61) <0.0001 |
| High | 383 | 829.33 (662.68, 995.97) <0.0001 | 720.68 (587.99, 853.37) <0.0001 |
| Thoracic/abdominal surgery |  |  |  |
| Yes |  |  |  |
| Low | 242 | 0 | 0 |
| Middle | 249 | 153.36 (-21.98, 328.69) 0.0869 | 89.26 (-47.76, 226.28) 0.2021 |
| High | 173 | 673.07 (479.68, 866.46) <0.0001 | 489.61 (338.48, 640.74) <0.0001 |
| No |  |  |  |
| Low | 785 | 0 | 0 |
| Middle | 1012 | 440.28 (346.69, 533.87) <0.0001 | 374.84 (297.93, 451.75) <0.0001 |
| High | 997 | 862.51 (768.62, 956.41) <0.0001 | 786.69 (709.52, 863.85) <0.0001 |
| Respiratory disease |  |  |  |
| Yes |  |  |  |
| Low | 220 | 0 | 0 |
| Middle | 219 | 477.13 (282.78, 671.49) <0.0001 | 375.59 (214.41, 536.77) <0.0001 |
| High | 163 | 825.46 (615.03, 1035.88) <0.0001 | 781.97 (607.47, 956.48) <0.0001 |
| No |  |  |  |
| Low | 807 | 0 | 0 |
| Middle | 1042 | 367.92 (275.38, 460.45) <0.0001 | 308.14 (232.60, 383.67) <0.0001 |
| High | 1007 | 847.19 (753.95, 940.43) <0.0001 | 742.87 (666.77, 818.98) <0.0001 |
| Cigarette |  |  |  |
| Yes |  |  |  |
| Low | 30 | 0 | 0 |
| Middle | 29 | 687.70 (203.11, 1172.28) 0.0067 | 638.61 (218.42, 1058.79) 0.0038 |
| High | 26 | 1137.31 (638.71, 1635.90) <0.0001 | 1149.74 (717.41, 1582.07) <0.0001 |
| No |  |  |  |
| Low | 997 | 0 | 0 |
| Middle | 1232 | 385.73 (301.15, 470.30) <0.0001 | 317.48 (248.29, 386.67) <0.0001 |
| High | 1144 | 847.69 (761.67, 933.71) <0.0001 | 748.77 (678.40, 819.14) <0.0001 |
| Weight (kg) Tertile |  |  |  |
| Low |  |  |  |
| Low | 248 | 0 | 0 |
| Middle | 430 | 66.17 (-62.21, 194.56) 0.3126 | 131.11 (19.61, 242.62) 0.0214 |
| High | 466 | 447.13 (320.57, 573.68) <0.0001 | 503.40 (393.48, 613.31) <0.0001 |
| Middle |  |  |  |
| Low | 327 | 0 | 0 |
| Middle | 400 | 541.57 (395.44, 687.69) <0.0001 | 393.35 (274.43, 512.26) <0.0001 |
| High | 419 | 1162.92 (1018.29, 1307.55) <0.0001 | 963.64 (845.95, 1081.34) <0.0001 |
| High |  |  |  |
| Low | 440 | 0 | 0 |
| Middle | 427 | 643.68 (506.67, 780.70) <0.0001 | 512.71 (399.76, 625.66) <0.0001 |
| High | 284 | 1241.05 (1087.52, 1394.57) <0.0001 | 1024.16 (897.60, 1150.72) <0.0001 |
| Standing Height (cm) Tertile |  |  |  |
| Low |  |  |  |
| Low | 418 | 0 | 0 |
| Middle | 442 | 126.55 (41.65, 211.45) 0.0036 | 128.41 (52.88, 203.93) 0.0009 |
| High | 277 | 239.89 (143.48, 336.30) <0.0001 | 261.41 (175.65, 347.17) <0.0001 |
| Middle |  |  |  |
| Low | 363 | 0 | 0 |
| Middle | 430 | 296.04 (195.38, 396.69) <0.0001 | 260.66 (171.11, 350.21) <0.0001 |
| High | 360 | 593.75 (488.71, 698.79) <0.0001 | 571.44 (477.99, 664.89) <0.0001 |
| High |  |  |  |
| Low | 234 | 0 | 0 |
| Middle | 386 | 471.72 (327.98, 615.45) <0.0001 | 382.57 (257.40, 507.75) <0.0001 |
| High | 532 | 713.08 (576.99, 849.17) <0.0001 | 686.37 (567.86, 804.89) <0.0001 |
| Systolic blood pressure (mmHg) Tertile |  |  |  |
| Low |  |  |  |
| Low | 288 | 0 | 0 |
| Middle | 402 | 250.13 (109.65, 390.60) 0.0005 | 201.13 (87.10, 315.17) 0.0006 |
| High | 362 | 621.38 (477.70, 765.06) <0.0001 | 553.28 (436.64, 669.92) <0.0001 |
| Middle |  |  |  |
| Low | 301 | 0 | 0 |
| Middle | 397 | 449.78 (295.81, 603.74) <0.0001 | 375.24 (249.65, 500.83) <0.0001 |
| High | 407 | 931.27 (778.12, 1084.41) <0.0001 | 823.23 (698.31, 948.15) <0.0001 |
| High |  |  |  |
| Low | 395 | 0 | 0 |
| Middle | 412 | 390.17 (245.38, 534.97) <0.0001 | 310.68 (191.92, 429.44) <0.0001 |
| High | 353 | 870.07 (719.47, 1020.67) <0.0001 | 765.75 (642.23, 889.27) <0.0001 |
| Diastolic blood pressure (mmHg) Tertile |  |  |  |
| Low |  |  |  |
| Low | 295 | 0 | 0 |
| Middle | 352 | 431.62 (276.10, 587.14) <0.0001 | 336.49 (206.24, 466.74) <0.0001 |
| High | 344 | 831.53 (675.19, 987.87) <0.0001 | 772.90 (641.96, 903.84) <0.0001 |
| Middle |  |  |  |
| Low | 350 | 0 | 0 |
| Middle | 459 | 287.31 (143.14, 431.48) <0.0001 | 252.35 (134.78, 369.91) <0.0001 |
| High | 402 | 776.61 (628.08, 925.14) <0.0001 | 693.64 (572.52, 814.76) <0.0001 |
| High |  |  |  |
| Low | 339 | 0 | 0 |
| Middle | 400 | 427.38 (283.55, 571.20) <0.0001 | 357.12 (240.42, 473.82) <0.0001 |
| High | 376 | 896.16 (750.24, 1042.08) <0.0001 | 764.45 (646.05, 882.84) <0.0001 |
| Glucose, serum (mmol/L) Tertile |  |  |  |
| Low |  |  |  |
| Low | 282 | 0 | 0 |
| Middle | 390 | 413.18 (262.65, 563.70) <0.0001 | 318.64 (196.96, 440.32) <0.0001 |
| High | 442 | 864.80 (718.04, 1011.57) <0.0001 | 762.00 (643.36, 880.64) <0.0001 |
| Middle |  |  |  |
| Low | 300 | 0 | 0 |
| Middle | 416 | 310.87 (157.70, 464.05) <0.0001 | 262.76 (136.64, 388.88) <0.0001 |
| High | 405 | 862.19 (708.14, 1016.23) <0.0001 | 750.16 (623.33, 877.00) <0.0001 |
| High |  |  |  |
| Low | 445 | 0 | 0 |
| Middle | 455 | 416.89 (284.39, 549.39) <0.0001 | 347.61 (239.81, 455.42) <0.0001 |
| High | 323 | 742.17 (596.90, 887.45) <0.0001 | 648.90 (530.70, 767.10) <0.0001 |
| Cholesterol (mmol/L) Tertile |  |  |  |
| Low |  |  |  |
| Low | 351 | 0 | 0 |
| Middle | 423 | 450.42 (305.54, 595.29) <0.0001 | 422.50 (304.04, 540.96) <0.0001 |
| High | 358 | 1006.72 (856.01, 1157.44) <0.0001 | 952.43 (829.19, 1075.67) <0.0001 |
| Middle |  |  |  |
| Low | 364 | 0 | 0 |
| Middle | 429 | 273.28 (133.88, 412.67) 0.0001 | 223.50 (108.19, 338.81) 0.0002 |
| High | 370 | 796.81 (652.40, 941.21) <0.0001 | 738.22 (618.77, 857.68) <0.0001 |
| High |  |  |  |
| Low | 312 | 0 | 0 |
| Middle | 408 | 470.90 (324.34, 617.46) <0.0001 | 342.86 (226.07, 459.64) <0.0001 |
| High | 442 | 827.49 (683.39, 971.59) <0.0001 | 656.22 (541.40, 771.05) <0.0001 |
| Creatinine (umol/L) Tertile |  |  |  |
| Low |  |  |  |
| Low | 391 | 0 | 0 |
| Middle | 435 | 3.29 (-108.09, 114.67) 0.9539 | 22.18 (-68.79, 113.14) 0.6329 |
| High | 295 | 353.37 (230.11, 476.63) <0.0001 | 360.29 (259.63, 460.96) <0.0001 |
| Middle |  |  |  |
| Low | 347 | 0 | 0 |
| Middle | 408 | 501.06 (354.81, 647.30) <0.0001 | 387.86 (266.29, 509.43) <0.0001 |
| High | 409 | 922.21 (776.05, 1068.37) <0.0001 | 794.01 (672.51, 915.51) <0.0001 |
| High |  |  |  |
| Low | 289 | 0 | 0 |
| Middle | 418 | 650.09 (499.42, 800.76) <0.0001 | 562.63 (435.84, 689.42) <0.0001 |
| High | 466 | 1010.33 (862.87, 1157.79) <0.0001 | 932.41 (808.32, 1056.51) <0.0001 |
| Alanine aminotransferase ALT (U/L) Tertile |  |  |  |
| Low |  |  |  |
| Low | 426 | 0 | 0 |
| Middle | 385 | 196.08 (72.04, 320.13) 0.0020 | 199.45 (95.29, 303.60) 0.0002 |
| High | 329 | 682.19 (552.72, 811.67) <0.0001 | 664.56 (555.85, 773.27) <0.0001 |
| Middle |  |  |  |
| Low | 318 | 0 | 0 |
| Middle | 445 | 400.57 (251.90, 549.25) <0.0001 | 336.40 (214.39, 458.41) <0.0001 |
| High | 351 | 816.47 (659.72, 973.23) <0.0001 | 744.92 (616.27, 873.56) <0.0001 |
| High |  |  |  |
| Low | 283 | 0 | 0 |
| Middle | 429 | 498.44 (341.05, 655.83) <0.0001 | 384.67 (256.32, 513.02) <0.0001 |
| High | 490 | 913.34 (759.89, 1066.79) <0.0001 | 768.29 (643.15, 893.42) <0.0001 |
| Total calcium (mmol/L) Tertile |  |  |  |
| Low |  |  |  |
| Low | 465 | 0 | 0 |
| Middle | 276 | 398.72 (260.18, 537.26) <0.0001 | 322.97 (212.87, 433.07) <0.0001 |
| High | 71 | 692.34 (460.04, 924.65) <0.0001 | 613.23 (428.60, 797.86) <0.0001 |
| Middle |  |  |  |
| Low | 374 | 0 | 0 |
| Middle | 509 | 442.68 (309.14, 576.22) <0.0001 | 349.77 (241.42, 458.11) <0.0001 |
| High | 348 | 926.69 (780.66, 1072.73) <0.0001 | 804.45 (685.96, 922.93) <0.0001 |
| High |  |  |  |
| Low | 188 | 0 | 0 |
| Middle | 475 | 514.44 (335.16, 693.73) <0.0001 | 441.71 (291.85, 591.57) <0.0001 |
| High | 751 | 1053.34 (883.65, 1223.03) <0.0001 | 926.45 (784.61, 1068.29) <0.0001 |
| Sodium (mmol/L) Tertile |  |  |  |
| Low |  |  |  |
| Low | 247 | 0 | 0 |
| Middle | 253 | 383.24 (206.45, 560.02) <0.0001 | 304.16 (163.96, 444.36) <0.0001 |
| High | 221 | 762.75 (579.75, 945.75) <0.0001 | 677.41 (532.28, 822.53) <0.0001 |
| Middle |  |  |  |
| Low | 390 | 0 | 0 |
| Middle | 494 | 361.02 (228.23, 493.81) <0.0001 | 305.18 (196.00, 414.37) <0.0001 |
| High | 474 | 793.82 (659.80, 927.85) <0.0001 | 704.90 (594.70, 815.09) <0.0001 |
| High |  |  |  |
| Low | 390 | 0 | 0 |
| Middle | 513 | 430.88 (295.71, 566.04) <0.0001 | 358.53 (246.85, 470.22) <0.0001 |
| High | 475 | 959.21 (821.73, 1096.69) <0.0001 | 850.73 (737.13, 964.32) <0.0001 |
| Potassium (mmol/L) Tertile |  |  |  |
| Low |  |  |  |
| Low | 290 | 0 | 0 |
| Middle | 348 | 265.78 (110.92, 420.63) 0.0008 | 260.16 (134.43, 385.88) <0.0001 |
| High | 385 | 728.04 (576.61, 879.47) <0.0001 | 685.68 (562.73, 808.62) <0.0001 |
| Middle |  |  |  |
| Low | 274 | 0 | 0 |
| Middle | 340 | 430.59 (273.04, 588.14) <0.0001 | 351.11 (219.97, 482.24) <0.0001 |
| High | 330 | 1049.40 (890.79, 1208.01) <0.0001 | 917.24 (785.22, 1049.26) <0.0001 |
| High |  |  |  |
| Low | 463 | 0 | 0 |
| Middle | 572 | 443.23 (317.09, 569.36) <0.0001 | 347.53 (244.27, 450.78) <0.0001 |
| High | 455 | 831.46 (698.26, 964.65) <0.0001 | 712.31 (603.27, 821.34) <0.0001 |

Note: (a) Including Multi-Racial; (b)Includes 12th grade with no diploma; (c) GED or equivalent. Weighted by: Full sample mobile examination center exam weight.
