# Supplementary material for: Supplementation of serum albumin is associated with improved pulmonary function: NHANES 2013–2014
Source: Front Physiol. 2022 Oct 3;13:948370. doi: 10.3389/fphys.2022.948370 (PMC9574070; doi:10.3389/fphys.2022.948370)
Supplement: Supplementary file 2 [file Table7.DOCX]

**Table S7. Analysis of threshold effect and saturation effect (Stratification by Thoracic/abdominal surgery).**

| **Baseline FVC** | **Thoracic/abdominal surgery** | **Yes**  **β(95%CI) *P*-value** | **No**  **β(95%CI) *P*-value** | **Total**  **β(95%CI) *P*-value** |
| --- | --- | --- | --- | --- |
|  | **Model I** |  |  | P-interaction: 0.112 |
|  | A straight-line effect | 138.66 (-11.06, 288.38) 0.0700 | 71.46 (-6.59, 149.51) 0.0729 | 80.40 (11.18, 149.61) 0.0229 |
|  | **Model II** |  |  | P-interaction: 0.227 |
|  | Fold points (K) | 4.5 | 3.8 | 4.5 |
|  | < K-segment effect 1 | 216.43 (28.33, 404.53) 0.0245 | -138.07 (-507.65, 231.52) 0.4641 | 100.26 (8.69, 191.83) 0.0320 |
|  | >K-segment Effect 2 | -159.35 (-621.13, 302.43) 0.4991 | 88.50 (5.11, 171.89) 0.0376 | 29.65 (-138.44, 197.74) 0.7296 |
|  | Effect size difference of 2 versus 1 | -375.78 (-926.65, 175.09) 0.1817 | 226.56 (-164.06, 617.18) 0.2557 | -70.61 (-283.74, 142.52) 0.5161 |
|  | Equation predicted values at break points | 3704.62 (3571.58, 3837.66) | 3408.88 (3329.46, 3488.30) | 4140.47 (4083.30, 4197.63) |
|  | Log likelihood ratio tests | 0.173 | 0.254 | 0.515 |
| **Baseline FEV 1** | **Thoracic/abdominal surgery** | **Yes**  **β(95%CI) *P*-value** | **No**  **β(95%CI) *P*-value** | **Total**  **β(95%CI) *P*-value** |
|  | **Model I** |  |  | P-interaction: 0.463 |
|  | A straight-line effect | 160.38 (28.63, 292.13) 0.0173 | 178.16 (109.84, 246.48) <0.0001 | 178.60 (117.92, 239.27) <0.0001 |
|  | **Model II** |  |  | P-interaction: 0.287 |
|  | Fold points (K) | 4.5 | 3.8 | 3.8 |
|  | < K-segment effect 1 | 234.51 (69.02, 400.00) 0.0056 | -205.56 (-528.81, 117.70) 0.2127 | -133.94 (-424.38, 156.49) 0.3661 |
|  | >K-segment Effect 2 | -123.69 (-529.95, 282.56) 0.5509 | 209.36 (136.42, 282.30) <0.0001 | 205.55 (140.15, 270.95) <0.0001 |
|  | Effect size difference of 2 versus 1 | -358.20 (-842.84, 126.43) 0.1480 | 414.92 (73.26, 756.57) 0.0174 | 339.50 (30.96, 648.03) 0.0311 |
|  | Equation predicted values at break points | 2864.52 (2759.97, 2969.06) | 2628.74 (2563.60, 2693.87) | 2571.94 (2513.61, 2630.28) |
|  | Log likelihood ratio tests | 0.14 | 0.017 | 0.03 |

Note: Abbreviations: FVC: forced vital capacity; FEV1: Forced expiratory volume in one second. Outcome variable: Baseline FVC (mL); Baseline FEV 1 (mL) ;Exposure variable: Albumin (g/dL) (mmol/L).Ajust: Age (years); Gender; Race/Hispanic origin; Education level; Respiratory disease; Cigarette; Weight (kg); Standing Height (cm); Systolic blood pressure (mmHg); Diastolic blood pressure (mmHg); Glucose, serum (mmol/L); Cholesterol (mmol/L); Creatinine (umol/L); Alanine aminotransferase ALT (U/L); Globulin (g/dL). When P < 0.05 in Model I, the model showed a Straight-line effect. When P > 0.05 in Model I, the model showed a segmented effect in Model II, with the K value being the serum albumin level at the fold point; β represents the slope of the curve, β for segments with P < 0.05 was statistically significant. The K value is the inflection point value, which is the level of serum albumin content at which the relationship between serum albumin and lung function changes.
