# Supplementary material for: Supplementation of serum albumin is associated with improved pulmonary function: NHANES 2013–2014
Source: Front Physiol. 2022 Oct 3;13:948370. doi: 10.3389/fphys.2022.948370 (PMC9574070; doi:10.3389/fphys.2022.948370)
Supplement: Supplementary file 3 [file Table2.DOCX]

**Table S2. Analysis of threshold effect and saturation effect (Stratification by gender).**

| **Baseline FVC** | **Gender** | **Male**  **β(95%CI) *P*-value** | **Female**  **β(95%CI) *P*-value** | **Total**  **β(95%CI) *P*-value** |
| --- | --- | --- | --- | --- |
|  | **Model I** |  |  | P-interaction: 0.137 |
|  | A straight-line effect | 54.24 (-57.86, 166.33) 0.3431 | 65.80 (-14.32, 145.92) 0.1077 | 80.40 (11.18, 149.61) 0.0229 |
|  | **Model II** |  |  | P-interaction: 0.180 |
|  | Fold points (K) | 4.5 | 4 | 4.5 |
|  | < K-segment effect 1 | 130.65 (-32.51, 293.82) 0.1167 | 144.26 (-66.98, 355.50) 0.1809 | 100.26 (8.69, 191.83) 0.0320 |
|  | >K-segment Effect 2 | -73.17 (-300.43, 154.09) 0.5281 | 40.32 (-61.90, 142.55) 0.4396 | 29.65 (-138.44, 197.74) 0.7296 |
|  | Effect size difference of 2 versus 1 | -203.82 (-520.11, 112.46) 0.2067 | -103.94 (-362.86, 154.98) 0.4315 | -70.61 (-283.74, 142.52) 0.5161 |
|  | Equation predicted values at break points | 4670.42 (4601.06, 4739.79) | 3213.14 (3155.85, 3270.43) | 4140.47 (4083.30, 4197.63) |
|  | Log likelihood ratio tests | 0.203 | 0.428 | 0.515 |
| **Baseline FEV 1** | **Gender** | **Male**  **β(95%CI) *P*-value** | **Female**  **β(95%CI) *P*-value** | **Total**  **β(95%CI) *P*-value** |
|  | **Model I** |  |  | P-interaction: 0.013 |
|  | A straight-line effect | 163.02 (64.09, 261.95) 0.0013 | 137.39 (68.41, 206.37) <0.0001 | 178.60 (117.92, 239.27) <0.0001 |
|  | **Model II** |  |  | P-interaction: 0.041 |
|  | Fold points (K) | 3.9 | 4.6 | 3.8 |
|  | < K-segment effect 1 | -145.95 (-577.75, 285.84) 0.5077 | 147.47 (68.66, 226.28) 0.0003 | -133.94 (-424.38, 156.49) 0.3661 |
|  | >K-segment Effect 2 | 191.82 (85.45, 298.20) 0.0004 | 53.73 (-269.84, 377.29) 0.7449 | 205.55 (140.15, 270.95) <0.0001 |
|  | Effect size difference of 2 versus 1 | 337.78 (-121.71, 797.27) 0.1498 | -93.74 (-447.96, 260.47) 0.6040 | 339.50 (30.96, 648.03) 0.0311 |
|  | Equation predicted values at break points | 3128.41 (3049.82, 3207.00) | 2746.87 (2690.03, 2803.70) | 2571.94 (2513.61, 2630.28) |
|  | Log likelihood ratio tests | 0.147 | 0.601 | 0.03 |

Note: Abbreviations: FVC: forced vital capacity; FEV1: Forced expiratory volume in one second.Outcome variable: Baseline FVC (mL); Baseline FEV 1 (mL) ;Exposure variable: Albumin (g/dL) (mmol/L).Ajust:Age (years); Race/Hispanic origin; Education level; Thoracic/abdominal surgery; Respiratory disease; Cigarette; Weight (kg); Standing Height (cm); Systolic blood pressure (mmHg); Diastolic blood pressure (mmHg); Glucose, serum (mmol/L); Cholesterol (mmol/L); Creatinine (umol/L); Alanine aminotransferase ALT (U/L); Globulin (g/dL). When P < 0.05 in Model I, the model showed a Straight-line effect. When P > 0.05 in Model I, the model showed a segmented effect in Model II, with the K value being the serum albumin level at the fold point; β represents the slope of the curve, β for segments with P < 0.05 was statistically significant. The K value is the inflection point value, which is the level of serum albumin content at which the relationship between serum albumin and lung function changes.
