# Supplementary material for: Supplementation of serum albumin is associated with improved pulmonary function: NHANES 2013–2014
Source: Front Physiol. 2022 Oct 3;13:948370. doi: 10.3389/fphys.2022.948370 (PMC9574070; doi:10.3389/fphys.2022.948370)
Supplement: Supplementary file 4 [file Table5.DOCX]

**Table S5. Analysis of threshold effect and saturation effect (Stratification by Cigarette).**

| **Baseline FVC** | **Cigarette** | **Yes**  **β(95%CI) *P*-value** | **No**  **β(95%CI) *P*-value** | **Total**  **β(95%CI) *P*-value** |
| --- | --- | --- | --- | --- |
|  | **Model I** |  |  | P-interaction: 0.450 |
|  | A straight-line effect | -44.07 (-593.23, 505.10) 0.8756 | 80.95 (11.11, 150.79) 0.0232 | 80.40 (11.18, 149.61) 0.0229 |
|  | **Model II** |  |  | P-interaction: 0.386 |
|  | Fold points (K) | 3.8 | 4.5 | 4.5 |
|  | < K-segment effect 1 | -2503.34 (-5898.53, 891.85) 0.1538 | 96.55 (4.15, 188.94) 0.0406 | 100.26 (8.69, 191.83) 0.0320 |
|  | >K-segment Effect 2 | 167.34 (-448.46, 783.14) 0.5963 | 41.01 (-128.90, 210.93) 0.6362 | 29.65 (-138.44, 197.74) 0.7296 |
|  | Effect size difference of 2 versus 1 | 2670.68 (-968.69, 6310.04) 0.1557 | -55.54 (-270.91, 159.84) 0.6133 | -70.61 (-283.74, 142.52) 0.5161 |
|  | Equation predicted values at break points | 3498.64 (3095.84, 3901.45) | 4129.87 (4072.06, 4187.68) | 4140.47 (4083.30, 4197.63) |
|  | Log likelihood ratio tests | 0.09 | 0.612 | 0.515 |
| **Baseline FEV 1** | **Cigarette** | **Yes**  **β(95%CI) *P*-value** | **No**  **β(95%CI) *P*-value** | **Total**  **β(95%CI) *P*-value** |
|  | **Model I** |  |  | P-interaction: 0.263 |
|  | A straight-line effect | 51.55 (-437.69, 540.79) 0.8371 | 178.72 (117.44, 239.99) <0.0001 | 178.60 (117.92, 239.27) <0.0001 |
|  | **Model II** |  |  | P-interaction: 0.022 |
|  | Fold points (K) | 3.8 | 3.8 | 3.8 |
|  | < K-segment effect 1 | -3577.38 (-6506.52, -648.24) 0.0199 | -84.98 (-376.85, 206.88) 0.5682 | -133.94 (-424.38, 156.49) 0.3661 |
|  | >K-segment Effect 2 | 363.50 (-167.77, 894.77) 0.1851 | 201.53 (135.49, 267.57) <0.0001 | 205.55 (140.15, 270.95) <0.0001 |
|  | Effect size difference of 2 versus 1 | 3940.88 (801.08, 7080.68) 0.0169 | 286.51 (-23.54, 596.56) 0.0702 | 339.50 (30.96, 648.03) 0.0311 |
|  | Equation predicted values at break points | 2533.37 (2185.19, 2881.56) | 2573.02 (2513.89, 2632.14) | 2571.94 (2513.61, 2630.28) |
|  | Log likelihood ratio tests | 0.004 | 0.069 | 0.03 |

Note: Abbreviations: FVC: forced vital capacity; FEV1: Forced expiratory volume in one second. Outcome variable: Baseline FVC (mL); Baseline FEV 1 (mL); Exposure variable: Albumin (g/dL) (mmol/L). Ajust: Age (years); Gender; Race/Hispanic origin; Education level; Thoracic/abdominal surgery; Respiratory disease; Weight (kg); Standing Height (cm); Systolic blood pressure (mmHg); Diastolic blood pressure (mmHg); Glucose, serum (mmol/L); Cholesterol (mmol/L); Creatinine (umol/L); Alanine aminotransferase ALT (U/L); Globulin (g/dL). When P < 0.05 in Model I, the model showed a Straight-line effect. When P > 0.05 in Model I, the model showed a segmented effect in Model II, with the K value being the serum albumin level at the fold point; β represents the slope of the curve, β for segments with P < 0.05 was statistically significant. The K value is the inflection point value, which is the level of serum albumin content at which the relationship between serum albumin and lung function changes.
