# Supplementary material for: Supplementation of serum albumin is associated with improved pulmonary function: NHANES 2013–2014
Source: Front Physiol. 2022 Oct 3;13:948370. doi: 10.3389/fphys.2022.948370 (PMC9574070; doi:10.3389/fphys.2022.948370)
Supplement: Supplementary file 5 [file Table3.DOCX]

**Table S3. Analysis of threshold effect and saturation effect (Stratification by age).**

| **Baseline FVC** | **Age (years) group** | **<60**  **β(95%CI) *P*-value** | **>=60**  **β(95%CI) *P*-value** | **Total**  **β(95%CI) *P*-value** |
| --- | --- | --- | --- | --- |
|  | **Model I** |  |  | P-interaction: 0.260 |
|  | A straight-line effect | 236.20 (156.51, 315.90) <0.0001 | 31.80 (-133.27, 196.87) 0.7059 | 209.76 (138.60, 280.92) <0.0001 |
|  | **Model II** |  |  | P-interaction: 0.610 |
|  | Fold points (K) | 4.6 | 3.9 | 3.8 |
|  | < K-segment effect 1 | 253.19 (152.77, 353.61) <0.0001 | -182.82 (-596.64, 230.99) 0.3869 | -46.96 (-394.10, 300.19) 0.7909 |
|  | >K-segment Effect 2 | 174.70 (-60.39, 409.80) 0.1454 | 96.72 (-104.31, 297.76) 0.3461 | 231.62 (154.81, 308.42) <0.0001 |
|  | Effect size difference of 2 versus 1 | -78.49 (-360.75, 203.77) 0.5858 | 279.55 (-214.74, 773.83) 0.2681 | 278.57 (-90.13, 647.28) 0.1387 |
|  | Equation predicted values at break points | 4434.42 (4370.38, 4498.45) | 3150.05 (3008.38, 3291.73) | 3331.39 (3260.10, 3402.69) |
|  | Log likelihood ratio tests | 0.584 | 0.258 | 0.137 |
| **Baseline FEV 1** | **Age (years) group** | **<60**  **β(95%CI) *P*-value** | **>=60**  **β(95%CI) *P*-value** | **Total**  **β(95%CI) *P*-value** |
|  | **Model I** |  |  | P-interaction: 0.006 |
|  | A straight-line effect | 375.12 (301.32, 448.92) <0.0001 | 74.59 (-74.13, 223.31) 0.3260 | 346.35 (280.43, 412.26) <0.0001 |
|  | **Model II** |  |  | P-interaction: 0.075 |
|  | Fold points (K) | 4.2 | 3.8 | 4.2 |
|  | < K-segment effect 1 | 243.54 (83.22, 403.86) 0.0029 | -236.73 (-664.12, 190.66) 0.2781 | 196.41 (61.67, 331.16) 0.0043 |
|  | >K-segment Effect 2 | 440.05 (338.19, 541.90) <0.0001 | 142.33 (-29.92, 314.58) 0.1059 | 431.75 (337.83, 525.68) <0.0001 |
|  | Effect size difference of 2 versus 1 | 196.50 (-16.07, 409.07) 0.0701 | 379.06 (-108.89, 867.00) 0.1284 | 235.34 (50.82, 419.86) 0.0125 |
|  | Equation predicted values at break points | 3124.15 (3075.36, 3172.94) | 2292.54 (2175.14, 2409.94) | 2956.37 (2911.13, 3001.60) |
|  | Log likelihood ratio tests | 0.069 | 0.121 | 0.012 |

Note: Abbreviations: FVC: forced vital capacity; FEV1: Forced expiratory volume in one second.Outcome variable: Baseline FVC (mL); Baseline FEV 1 (mL) ;Exposure variable: Albumin (g/dL) (mmol/L).Ajust: Gender; Race/Hispanic origin; Education level; Thoracic/abdominal surgery; Respiratory disease; Cigarette; Weight (kg); Standing Height (cm); Systolic blood pressure (mmHg); Diastolic blood pressure (mmHg); Glucose, serum (mmol/L); Cholesterol (mmol/L); Creatinine (umol/L); Alanine aminotransferase ALT (U/L); Globulin (g/dL). When P < 0.05 in Model I, the model showed a Straight-line effect. When P > 0.05 in Model I, the model showed a segmented effect in Model II, with the K value being the serum albumin level at the fold point; β represents the slope of the curve, β for segments with P < 0.05 was statistically significant. The K value is the inflection point value, which is the level of serum albumin content at which the relationship between serum albumin and lung function changes.
