# Supplementary material for: Supplementation of serum albumin is associated with improved pulmonary function: NHANES 2013–2014
Source: Front Physiol. 2022 Oct 3;13:948370. doi: 10.3389/fphys.2022.948370 (PMC9574070; doi:10.3389/fphys.2022.948370)
Supplement: Supplementary file 6 [file Table4.DOCX]

**Table S4. Analysis of threshold effect and saturation effect (Stratification by Race/Hispanic origin).**

| **Baseline FVC** | **Race/Hispanic origin** | **Mexican American**  **β(95%CI) *P*-value** | **Other Hispanic**  **β(95%CI) *P*-value** | **Non-Hispanic white**  **β(95%CI) *P*-value** | **Non-Hispanic black**  **β(95%CI) *P*-value** | **Other races - Including multi-racial**  **β(95%CI) *P*-value** | **Total**  **β(95%CI) *P*-value** |
| --- | --- | --- | --- | --- | --- | --- | --- |
|  | **Model I** |  |  |  |  |  | P-interaction: 0.019 |
|  | A straight-line effect | 120.19 (-77.14, 317.51) 0.2334 | 8.45 (-204.18, 221.08) 0.9379 | 105.64 (-19.39, 230.67) 0.0980 | 127.80 (0.84, 254.75) 0.0488 | 48.20 (-118.31, 214.71) 0.5707 | 80.40 (11.18, 149.61) 0.0229 |
|  | **Model II** |  |  |  |  |  | P-interaction: 0.128 |
|  | Fold points (K) | 4.1 | 4.6 | 4 | 3.8 | 4.2 | 4.5 |
|  | < K-segment effect 1 | 310.88 (-188.36, 810.12) 0.2231 | 81.08 (-175.82, 337.97) 0.5366 | 381.78 (-85.10, 848.67) 0.1093 | -214.23 (-649.39, 220.93) 0.3349 | 213.44 (-191.97, 618.84) 0.3026 | 100.26 (8.69, 191.83) 0.0320 |
|  | >K-segment Effect 2 | 59.39 (-186.26, 305.05) 0.6359 | -354.68 (-1106.16, 396.80) 0.3556 | 61.44 (-82.83, 205.70) 0.4041 | 187.92 (41.49, 334.35) 0.0121 | -11.21 (-224.29, 201.86) 0.9179 | 29.65 (-138.44, 197.74) 0.7296 |
|  | Effect size difference of 2 versus 1 | -251.49 (-856.21, 353.24) 0.4156 | -435.76 (-1300.67, 429.15) 0.3242 | -320.35 (-842.19, 201.50) 0.2292 | 402.15 (-87.29, 891.59) 0.1077 | -224.65 (-727.17, 277.87) 0.3813 | -70.61 (-283.74, 142.52) 0.5161 |
|  | Equation predicted values at break points | 3830.44 (3673.82, 3987.07) | 4144.70 (3960.82, 4328.58) | 3904.61 (3797.97, 4011.24) | 3036.17 (2924.58, 3147.76) | 3583.78 (3453.54, 3714.03) | 4140.47 (4083.30, 4197.63) |
|  | Log likelihood ratio tests | 0.401 | 0.308 | 0.225 | 0.103 | 0.372 | 0.515 |
| **Baseline FEV 1** | **Race/Hispanic origin** | **Mexican American**  **β(95%CI) *P*-value** | **Other Hispanic**  **β(95%CI) *P*-value** | **Non-Hispanic white**  **β(95%CI) *P*-value** | **Non-Hispanic black**  **β(95%CI) *P*-value** | **Other races - Including multi-racial**  **β(95%CI) *P*-value** | **Total**  **β(95%CI) *P*-value** |
|  | **Model I** |  |  |  |  |  | P-interaction: 0.001 |
|  | A straight-line effect | 174.48 (21.52, 327.44) 0.0260 | 147.92 (-30.60, 326.44) 0.1054 | 266.81 (151.56, 382.05) <0.0001 | 155.27 (38.09, 272.45) 0.0096 | 86.11 (-42.88, 215.10) 0.1913 | 178.60 (117.92, 239.27) <0.0001 |
|  | **Model II** |  |  |  |  |  | P-interaction: 0.015 |
|  | Fold points (K) | 3.9 | 3.8 | 4 | 3.8 | 4.8 | 3.8 |
|  | < K-segment effect 1 | 4.06 (-538.91, 547.02) 0.9883 | -945.05 (-2483.73, 593.64) 0.2296 | 509.92 (79.55, 940.29) 0.0204 | -191.50 (-593.03, 210.03) 0.3502 | 44.19 (-100.23, 188.60) 0.5489 | -133.94 (-424.38, 156.49) 0.3661 |
|  | >K-segment Effect 2 | 196.56 (29.25, 363.87) 0.0219 | 201.29 (8.05, 394.53) 0.0420 | 227.89 (94.91, 360.87) 0.0008 | 216.22 (81.11, 351.34) 0.0018 | 487.73 (-149.01, 1124.47) 0.1338 | 205.55 (140.15, 270.95) <0.0001 |
|  | Effect size difference of 2 versus 1 | 192.51 (-395.92, 780.93) 0.5218 | 1146.34 (-456.62, 2749.30) 0.1620 | -282.03 (-763.06, 199.00) 0.2507 | 407.73 (-43.89, 859.34) 0.0772 | 443.54 (-245.09, 1132.18) 0.2073 | 339.50 (30.96, 648.03) 0.0311 |
|  | Equation predicted values at break points | 2876.27 (2729.99, 3022.55) | 2609.21 (2426.95, 2791.47) | 2942.41 (2852.77, 3032.04) | 2404.00 (2308.19, 2499.80) | 3307.83 (3187.45, 3428.22) | 2571.94 (2513.61, 2630.28) |
|  | Log likelihood ratio tests | 0.509 | 0.148 | 0.246 | 0.074 | 0.199 | 0.03 |

Note: Abbreviations: FVC: forced vital capacity; FEV1: Forced expiratory volume in one second. Outcome variable: Baseline FVC (mL); Baseline FEV 1 (mL) ;Exposure variable: Albumin (g/dL) (mmol/L).Ajust: Age (years); Gender; Education level; Thoracic/abdominal surgery; Respiratory disease; Cigarette; Weight (kg); Standing Height (cm); Systolic blood pressure (mmHg); Diastolic blood pressure (mmHg); Glucose, serum (mmol/L); Cholesterol (mmol/L); Creatinine (umol/L); Alanine aminotransferase ALT (U/L); Globulin (g/dL). When P < 0.05 in Model I, the model showed a Straight-line effect. When P > 0.05 in Model I, the model showed a segmented effect in Model II, with the K value being the serum albumin level at the fold point; β represents the slope of the curve, β for segments with P < 0.05 was statistically significant. The K value is the inflection point value, which is the level of serum albumin content at which the relationship between serum albumin and lung function changes.
