# Supplementary material for: Supplementation of serum albumin is associated with improved pulmonary function: NHANES 2013–2014
Source: Front Physiol. 2022 Oct 3;13:948370. doi: 10.3389/fphys.2022.948370 (PMC9574070; doi:10.3389/fphys.2022.948370)
Supplement: Supplementary file 7 [file Table6.DOCX]

**Table S6. Analysis of threshold effect and saturation effect (Stratification by Respiratory disease).**

| **Bseeline FVC** | **Respiratory disease** | **Yes**  **β(95%CI) *P*-value** | **No**  **β(95%CI) *P*-value** | **Total**  **β(95%CI) *P*-value** |
| --- | --- | --- | --- | --- |
|  | **Model I** |  |  | P-interaction: 0.314 |
|  | A straight-line effect | 95.18 (-71.24, 261.61) 0.2628 | 72.46 (-3.73, 148.66) 0.0624 | 80.40 (11.18, 149.61) 0.0229 |
|  | **Model II** |  |  | P-interaction: 0.601 |
|  | Fold points (K) | 3.8 | 4.6 | 4.5 |
|  | < K-segment effect 1 | -145.48 (-664.67, 373.71) 0.5831 | 110.89 (17.48, 204.31) 0.0201 | 100.26 (8.69, 191.83) 0.0320 |
|  | >K-segment Effect 2 | 135.45 (-50.22, 321.12) 0.1533 | -84.39 (-317.83, 149.06) 0.4787 | 29.65 (-138.44, 197.74) 0.7296 |
|  | Effect size difference of 2 versus 1 | 280.93 (-293.14, 855.00) 0.3379 | -195.28 (-470.01, 79.45) 0.1637 | -70.61 (-283.74, 142.52) 0.5161 |
|  | Equation predicted values at break points | 3290.69 (3127.26, 3454.13) | 4288.24 (4222.55, 4353.92) | 4140.47 (4083.30, 4197.63) |
|  | Log likelihood ratio tests | 0.327 | 0.162 | 0.515 |
| **Baseline FEV 1** | **Respiratory disease** | **Yes**  **β(95%CI) *P*-value** | **No**  **β(95%CI) *P*-value** | **Total**  **β(95%CI) *P*-value** |
|  | **Model I** |  |  | P-interaction: 0.256 |
|  | A straight-line effect | 228.90 (73.89, 383.91) 0.0040 | 163.86 (98.00, 229.73) <0.0001 | 178.60 (117.92, 239.27) <0.0001 |
|  | **Model II** |  |  | P-interaction: 0.253 |
|  | Fold points (K) | 3.8 | 3.8 | 3.8 |
|  | < K-segment effect 1 | -172.57 (-655.24, 310.10) 0.4837 | -101.70 (-496.41, 293.02) 0.6136 | -133.94 (-424.38, 156.49) 0.3661 |
|  | >K-segment Effect 2 | 296.08 (123.47, 468.69) 0.0008 | 182.04 (111.00, 253.09) <0.0001 | 205.55 (140.15, 270.95) <0.0001 |
|  | Effect size difference of 2 versus 1 | 468.65 (-65.04, 1002.34) 0.0858 | 283.74 (-132.08, 699.56) 0.1812 | 339.50 (30.96, 648.03) 0.0311 |
|  | Equation predicted values at break points | 2496.88 (2360.52, 2633.25) | 2591.96 (2526.66, 2657.27) | 2571.94 (2513.61, 2630.28) |
|  | Log likelihood ratio tests | 0.079 | 0.179 | 0.03 |

Note: Abbreviations: FVC: forced vital capacity; FEV1: Forced expiratory volume in one second. Outcome variable: Baseline FVC (mL); Baseline FEV 1 (mL) ;Exposure variable: Albumin (g/dL) (mmol/L).Ajust: Age (years); Gender; Race/Hispanic origin; Education level; Thoracic/abdominal surgery; Cigarette; Weight (kg); Standing Height (cm); Systolic blood pressure (mmHg); Diastolic blood pressure (mmHg); Glucose, serum (mmol/L); Cholesterol (mmol/L); Creatinine (umol/L); Alanine aminotransferase ALT (U/L); Globulin (g/dL). When P < 0.05 in Model I, the model showed a Straight-line effect. When P > 0.05 in Model I, the model showed a segmented effect in Model II, with the K value being the serum albumin level at the fold point; β represents the slope of the curve, β for segments with P < 0.05 was statistically significant. The K value is the inflection point value, which is the level of serum albumin content at which the relationship between serum albumin and lung function changes.
